# Control of the Rescue and Replication of Semliki Forest Virus Recombinants by the Insertion of miRNA Target Sequences

**DOI:** 10.1371/journal.pone.0075802

**Published:** 2013-09-30

**Authors:** Kaspar Ratnik, Liane Viru, Andres Merits

**Affiliations:** Institute of Technology, University of Tartu, Tartu, Estonia; University of Pittsburgh School of Medicine, United States of America

## Abstract

Due to their broad cell- and tissue-tropism, alphavirus-based replication-competent vectors are of particular interest for anti-cancer therapy. These properties may, however, be potentially hazardous unless the virus infection is controlled. While the RNA genome of alphaviruses precludes the standard control techniques, host miRNAs can be used to down-regulate viral replication. In this study, target sites from ubiquitous miRNAs and those of miRNAs under-represented in cervical cancer cells were inserted into replication-competent DNA/RNA layered vectors of Semliki Forest virus. It was found that in order to achieve the most efficient suppression of recombinant virus rescue, the introduced target sequences must be fully complementary to those of the corresponding miRNAs. Target sites of ubiquitous miRNAs, introduced into the 3′ untranslated region of the viral vector, profoundly reduced the rescue of recombinant viruses. Insertion of the same miRNA targets into coding region of the viral vector was approximately 300-fold less effective. Viruses carrying these miRNAs were genetically unstable and rapidly lost the target sequences. This process was delayed, but not completely prevented, by miRNA inhibitors. Target sites of miRNA under-represented in cervical cancer cells had much smaller but still significant effects on recombinant virus rescue in cervical cancer-derived HeLa cells. Over-expression of *miR-214*, one of these miRNAs, reduced replication of the targeted virus. Though the majority of rescued viruses maintained the introduced miRNA target sequences, genomes with deletions of these sequences were also detected. Thus, the low-level repression of rescue and replication of targeted virus in HeLa cells was still sufficient to cause genetic instability.

## Introduction

Members of genus *Alphavirus* (family *Togaviridae*) are small, enveloped, positive-stranded RNA viruses [1]. Most alphaviruses are transmitted by mosquitoes and can infect many vertebrate hosts. Several alphaviruses infect humans. Recent outbreaks of Chikungunya virus affected millions of people in tropical regions of Africa and Asia, causing both health problems and ecological concerns [2]. The most studied alphaviruses are Venezuelan Equine Encephalitis virus (VEEV), Semliki Forest virus (SFV) and Sindbis virus (SINV). Attenuated strains of these viruses are apathogenic to humans and have been used as tools for basic research. In addition, many efficient gene expression and potential anti-cancer therapy vectors have been constructed from their genomes [3].

As alphavirus genomes are small and simply organised, the generation of alphavirus-based recombinant vectors is relatively straightforward. The 5′ two-thirds of the viral RNA genome contain an open reading frame (ORF) for four non-structural (ns) proteins (nsP1–4). nsPs are expressed as a ns-polyprotein precursor and represent components of viral replicase [4]. Structural proteins, which are not required for genome replication, are translated from a subgenomic (SG) mRNA that is generated by internal initiation from the SG promoter located on the complementary negative-strand RNA template. Both genomic RNA and SG mRNAs are synthesised in high numbers, usually exceeding 100,000 copies per cell. For vertebrate cells, alphavirus infection is cytotoxic and results in shutdown of host cell RNA and protein synthesis, leading ultimately to cell death [5]–[9]. Multiple mechanisms by which alphaviruses shut down synthesis of host cell RNAs and proteins and counter-act host cell antiviral defences are known [5], [9]–[11].

The alphavirus-based expression and gene therapy vectors take advantage of the viral genome structure. Frequently, the coding sequences of ns- and structural proteins are separated onto two different RNA molecules to generate an alphavirus replicon vector system [12]. Transfection of cells with *in vitro* synthesised replicon RNA, where the coding sequence of structural proteins is replaced with that of a foreign protein of interest, results in high-level expression of the latter (up to 25% of total cell protein). Replicon RNAs can also be efficiently packed into virus-replicon particles (VRPs, also called “suicide” particles), which can be used in further applications. In this method, a packaging-deficient helper-RNA that encodes only structural proteins is co-transfected with alphavirus replicon RNA [12], [13]. VRPs are safe and useful vectors, but, as they cannot be propagated, their production is costly. VRPs have also limited applications *in vivo*, as they cannot initiate a productive infection. For these reasons, replication-competent alphavirus vectors have been developed. These vectors are essentially recombinant viruses that are capable of multiple rounds of infection in cell culture or *in vivo*. These vectors are generated by the insertion of coding sequences from exogenous genes into alphavirus ORFs [14], [15] or into separate expression units that are usually under the control of a duplicated SG promoter [16] or a picornavirus internal ribosomal entry site (IRES) [17], [18]. The location of the inserted sequences and the mode of their expression can be used to regulate the quantity and timing of their expression.

Two different approaches are used to rescue infectious RNA genomes from recombinant plasmids containing viral cDNAs. The viral cDNAs are usually placed under the control of the SP6 or T7 RNA polymerase promoter so that infectious RNA can be produced by *in vitro* transcription. This RNA is subsequently used to transfect cells, in which viral replication and particle formation are initiated [12]. Alternatively, recombinant plasmids contain viral cDNAs under the control of a eukaryotic RNA polymerase II promoter, which is typically the immediate-early promoter of human cytomegalovirus (CMV). Upon delivery of such plasmid to the nucleus of transfected cell, cellular RNA polymerase drives transcription of infectious RNA, which is transported to cytoplasm where it initiates replication and virion formation [19], [20]. Due to this behaviour, such vectors are often referred to as DNA/RNA layered vectors.

Replication-competent alphavirus vectors have potential uses in anti-cancer therapy, as they can be engineered to express tumour antigens and/or anti-tumour proteins and can kill cancer cells [21]. Furthermore, alphavirus vectors stimulate the anti-cancer immune response via enhanced antigen expression and initiation of the apoptotic cascade; in this regard, alphavirus vectors are superior to the conventional anti-cancer DNA vaccines [22]. SINV is reported to possess a natural tropism for tumours [23]–[25]. Most alphaviruses, however, lack this property, illustrating the main argument against using alphaviruses (or other RNA viruses) as anti-cancer agents: there are no easy ways to restrict viral infection to the target cancer cells. Several approaches to achieve cell-specificity during alphavirus rescue and replication have been proposed by us [26] and others [27], but none of them absolutely protects non-tumour tissues. Therefore making the viral genomes sensitive to inhibition by pre-existing cellular miRNAs has been proposed as an approach which could restrict spread of infection [28], [29].

miRNAs are small RNAs that regulate posttranscriptional gene expression [30]. They are approximately 21 nucleotides in length and were discovered in *Caenorhabditis elegans* [31]. Hundreds of miRNAs have been identified by molecular cloning and bioinformatics [32], [33]. miRNAs bind mainly to sequences in the 3′ untranslated region (UTR) of target mRNA and induce inhibition of translation or mRNA degradation [30]. The most important region of miRNA is a 7-nucleotide seed region (nucleotides 2–8 at the 5′ end) [34]. Perfect base pairing between the seed-region and target sequence is essential for miRNA action. The effect is enhanced if a perfect 21-bp duplex is formed between the miRNA and its target. In this case, the targeted mRNA is cleaved by the Slicer activity of the Argonaute enzyme and degraded. The cellular miRNA pool depends on the cell type and developmental stage of cells and tissues. Notably, cancer cells exhibit distinct miRNA profiles with a general tendency that miRNA expression in tumours is globally reduced compared to normal tissues [35], [36].

These properties of miRNAs have been used in development of virus-based vectors. miRNA target sequences have been inserted into the genomes of vesicular stomatitis virus [37], coxsackievirus [38], poliovirus [39], chimeric tick-borne encephalitis/dengue virus [40], herpes simplex virus 1 [41] and several others. This genetic engineering generates viral tropism toward targeted tissues, limits or enhances viral immunogenicity, decreases cytotoxic side effects of virus infection and increases the safety of the constructed vectors.

Similar to other viruses, miRNA target sites have also been used to modify alphavirus-based vectors. First, a cluster of target sites of ubiquitously expressed miRNAs was inserted into the 5′ and/or 3′ UTR region of helper RNAs used for packaging VEEV replicon vectors. Obtained helper RNAs are unable to efficiently replicate in the absence of miRNA inhibitors (modified oligonucleotides, antisense to miRNA sequences). Similarly, recombinant viruses generated by recombination between replicon and helper RNAs were restricted in their replication, resulting in improved safety of the vector system [28]. In another study, six target sites for the neuron-specific miRNA *miR-124* were inserted into ns-protein ORF of SFV; the resulting virus replicates in peripheral tissues and oligodendrocytes, but was restricted from neurons and had remarkably reduced neurovirulence [29].

**Figure 1:**
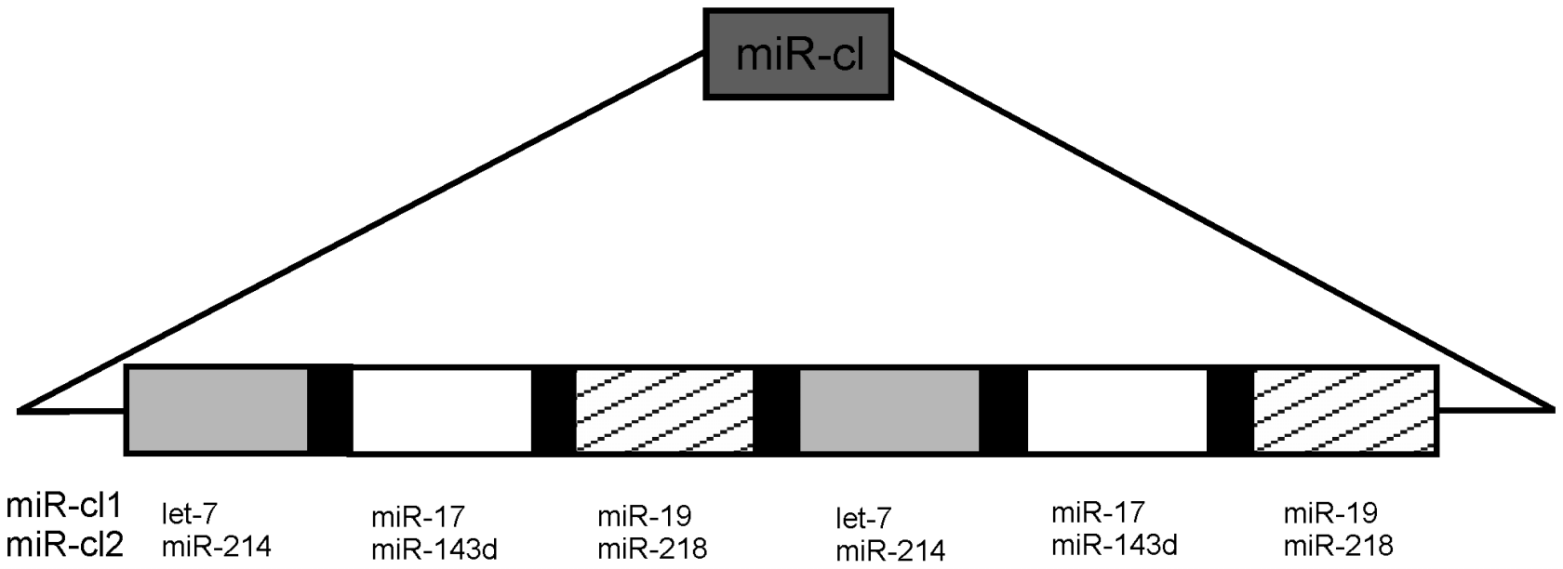
Illustration of used miR target cassette design. Identical miRNA target sites are shown using the same colour code (grey, white or striped); the 8-nucleotide spacers between miRNA target sites are shown in black.

To compare these approaches and analyse the limitations of use of miRNA targets in the rescue and replication of alphavirus vectors, a panel of DNA/RNA layered SFV replication-competent vectors was constructed. These vectors expressed a *Gaussia* luciferase (Gluc) reporter via a duplicated SG-promoter, which allowed detailed monitoring of viral infection and gene expression in cell culture. To analyse how different mechanisms of miRNA action can be used to restrict virus infection, three alternative strategies of miRNA binding were compared. miRNA target cassettes (Fig. 1) contained either naturally existing miRNA target sites, sites designed to form a perfect duplex with miRNA or sites that bound miRNA in a “sponge” configuration (Table 1). Targets that formed perfect duplexes with ubiquitously expressed cellular miRNAs had by far the highest impact on the infectivity of recombinant vectors. Such miRNA target site, inserted into 3′ UTR region of the SFV genome, had nearly 300-fold greater impact on the infectivity than the same target inserted into ns-protein ORF. Despite the inhibition of virus rescue, the recombinant viruses rapidly acquired the ability to grow to very high titre; this effect was associated with loss of the inserted miRNA target region. This process could be delayed, but not prevented, by miRNA inhibitors. Incorporation of target sites of miRNAs that are under-represented in cervical cancer cells had far less impact on the efficiency of recombinant virus rescue. These viruses replicated well in both cervical carcinoma-derived HeLa cells and control BHK-21 cells. Nevertheless, viruses that lost the engineered miRNA targets did emerge. This result indicates that the corresponding miRNAs were still present in quantities sufficient to cause selection against viruses containing their respective targets. Taken together, these results highlighted both the potential and the limitations of miRNA-based regulation of alphavirus vectors.

**Table 1:** Native, perfect and sponge target sequences used in miR target cassettes.

miRNA	Type	Sequence (5′ to 3′)	Reference
*Let7*	Native	CGCCAACGTTCGATTT**CTACCTC**A	[55]
*Let7*	Perfect	AACTATACAACCTA**CTACCTC**A	
*Let7*	Sponge	AACTATACAAGGAT **CTACCTC**A	
*miR-17*	Native	GTACCGTTGCATTAGA**GCACTTT**G	[56]
*miR-17*	Perfect	ACTACCTGCACTGTAA**GCACTTT**G	
*miR-17*	Sponge	ACTACCTGCACTCATT **GCACTTT**G	
*miR-19*	Native	AATGAGTTTTGCAGT**TTTGCAC**A	[57]
*miR-19*	Perfect	TCAGTTTTGCATGGA**TTTGCAC**A	
*miR-19*	Sponge	TCAGTTTTGCAACCT **TTTGCAC**A	
*miR-214*	Native	TTTCAATCATAATA**CCTGCTG**TG	[58]
*miR-214*	Perfect	ACTGCCTGTCTGTG**CCTGCTG**T	
*miR-214*	Sponge	ACTGCCTGTCCAGT **CCTGCTG**T	
*miR-143d*	Native	GTTACAGTTTGCACAAGT**TCATCTC**AT	[59]
*miR-143d*	Perfect	GAGCTACAGTGCT**TCATCTC**A	
*miR-143d*	Sponge	GAGCTACAGGTAC **TCATCTC**A	
*miR-218*	Native	CACTGGTGACTTGA**AAGCACA**A	[60]
*miR-218*	Perfect	ACATGGTTAGATC**AAGCACA**A	
*miR-218*	Sponge	ACATGGTTATCGT **AAGCACA**A	

Bold: sequences matching the seed region of miRNA.

Underlined: sequenced modified to generate sponge design.

## Materials and Methods

### Cell lines

All cells were cultured at 37°C in humidified 5% CO_2_ atmosphere. BHK-21 cells (ATCC-CCL-10) were cultured in Glasgow minimal essential medium (GMEM, Sigma-Aldrich) supplemented with 10% foetal calf serum (FCS, PAA), 20 mM HEPES (Sigma-Aldrich), 10% tryptose phosphate broth (Becton Dickinson) and penicillin-streptomycin (Invitrogen). HeLa cells (ATCC-CCL-2) were cultured in Iscove's modified Dulbecco's medium (IMDM, Sigma-Aldrich) supplemented with 10% FCS and penicillin-streptomycin. HeLa cells over-expressing *miR-214* were generated by transfection with a plasmid containing an *miR-214* precursor expression cassette (GeneCopoeia) and subsequent selection of puromycin-resistant colonies. The resultant cell line was designated as HeLa-miR214 and cultured in Iscove's modified Dulbecco's medium (Sigma-Aldrich) supplemented with 10% FCS, penicillin-streptomycin and 10 µg/ml puromycin (Life Technologies).

### miRNA cassette design

miRNA cassettes contained two copies of three selected miRNA target sequences (Table 1; Fig. 1). The target sequences were separated from each other by 8-nucleotide spacers with the sequence 5′ CTTAAATG 3′. For the cassette, which was subsequently inserted into the ns-region of the DNA/RNA layered SFV vector, the spacer with the sequence 5′ CTTATATG 3′ was used. This modification was essential to avoid in-frame stop codons that would disrupt ns-polyprotein translation. All cassettes were ordered as synthetic DNAs (Life Technologies).

### Cloning procedures

DNA/RNA layered SFV replication-competent vectors were constructed from the pCMV-SFV4 vector [20]. The sequence corresponding to the SG promoter of SFV4 (from position −37 to +17 with respect to the transcription start site) was inserted immediately downstream of the termination codon of the structural ORF [42]. The synthetic sequence encoding codon-optimised Gluc (Life Technologies) was inserted downstream of the duplicated SG-promoter; the resulting construct was designated as pCMV-SFV4-2SG-Gluc. To construct DNA/RNA layered vectors containing miR targets in their 3′ UTR region, the corresponding target sequences were inserted 6 nucleotides downstream of the Gluc terminator codon of pCMV-SFV4-2SG-Gluc vector. Vectors containing miR targets composed from sequences fully complementary to selected miRNAs were designated as pCMV-SFV4-2SG-Gluc-miR-cl1P and pCMV-SFV4-2SG-Gluc-miR-cl2P; vectors designated as pCMV-SFV4-2SG-Gluc-miR-cl1N and pCMV-SFV4-2SG-Gluc-miR-cl2N contained target sites of native design and vectors pCMV-SFV4-2SG-Gluc-miR-cl1S and pCMV-SFV4-2SG-Gluc-miR-cl2S contained target sites of sponge design. To obtain the DNA/RNA layered SFV replication-competent vectors containing an miR-cl1P-target cassette in the ns-region of the vector, a construct containing the coding sequence for ZsGreen followed by a miR-cl1P target flanked with *Asc*I cleavage sites (Life Technologies) was used. The sequence encoding for ZsGreen and the miR-cl1P-target cassette was inserted into pCMV-SFV4-2SG-Gluc as previously described [15], and the resultant vector was designated pCMV-SFV(nsZsGreen-miR-cl1P)4-2SG-Gluc. To obtain the control vector pCMV-SFV(nsZsGreen)4-2SG-Gluc, the miR-cl1P target sequence was removed via *Asc*I cleavage. Non-replicative plasmid vectors for expression of Gluc from the CMV promoter were obtained from pCMV-SFV4-2SG-Gluc and DNA/RNA layered SFV replication-competent vectors containing miR-target sites in the 3′ UTR region; all viral sequences except the 5′ and 3′ UTR were deleted by PCR-mediated mutagenesis. All clones were verified by Sanger sequencing. Detailed cloning procedures and sequences of all constructs are available upon request.

### Transfection procedures and infectious centre assay

All transfections were conducted by electroporation. For both BHK-21 and HeLa cells, the electroporation solution contained cytomix (120 mM KCl, 0.15 mM CaCl2, 10 mM K_2_HPO_4_/KH_2_HPO_4_, pH 7.6, 25 mM HEPES, pH 7.6, 2 mM EGTA, pH 7.6, 5 mM MgCl_2_ and 5 mM glutathione), 2 mM ATP, 0.5% NaBes and 50 µg herring sperm DNA. Electroporation was conducted with the following conditions: 220 V, 975 µF, one pulse in a cuvette with 0.4 cm electrode cap using a Gene Pulser II device (BioRad).

For infectious centre assays (ICA) 1 µg DNA/RNA layered SFV replication-competent vector was transfected into 8×10^6^ BHK-21, HeLa or HeLa-miR214 cells. Tenfold serial dilutions of electroporated cells were seeded on six-well tissue culture plates that contained 1.5×10^6^ BHK-21 cells per well. After 2 h incubation at 37°C, cells were overlaid with 2 ml carboxymethyl cellulose (CMC)-containing GMEM (final concentration of CMC 0.8%) supplemented with 1.2% FCS. Plaques were stained with crystal violet after 2 days incubation at 37°C. When needed, the remaining electroporated cells were seeded on a tissue culture plate and incubated for 1 h at 37°C, after which the media was replaced with cultivation media. Stocks of recombinant viruses were collected at 24 h post-transfection (p.t.).

To monitor virus rescue, accumulation and Gluc marker expression, 8×10^6^ BHK-21, HeLa or HeLa-miR214 cells were transfected as described above. To investigate the effect of 2-O-Met miRNA inhibitors on virus growth in BHK-21 cells, 300 pmol of each miRNA inhibitor (anti-*let7* 5′ AACUAUACAACCUACUACCUCA 3′; anti-*miR-17* 5′ ACUACCUGCACUGUAAGCACUUUG 3′; anti-*miR-19* 5′UACGUUUUGCAUAGAUUUGCACA 3′) or 900 pmol of irrelevant control oligonucleotide (5′ CCUCUUACCUCAGUUACA 3′) was added into the electroporation solution. Aliquots of growth media (200 µl) containing secreted Gluc and released virions were collected at selected time points. Gluc activity in growth media was assayed with a Renilla Luciferase Assay System and Glomax 20/20 Luminometer (Promega).

For analysis of Gluc expression by non-viral vectors, 150 ng of each plasmid was electroporated into 8×10^6^ BHK-21 or HeLa cells. To measure Gluc activity, aliquots of growth media were collected at selected times p.t..

### Virus titration and analysis of homogeneity of viral stocks

Virus stocks were quantified by plaque assay. Tenfold serial dilutions of virus stocks were used to infect confluent monolayers of BHK-21 cells. After 1 h incubation at 37°C, the inoculum was removed, and cells were covered with 2 ml CMC-containing GMEM supplemented with 1.2% FCS. Plaques were stained with crystal violet after at 2 days post-infection (p.i.). The growth curves of replication-competent SFV4 vectors were analysed as previously described [42].

For analysis of genetic stability of recombinant viruses the collected virus stocks were propagated at a multiplicity of infection (MOI) of 0.1 plaque-forming units (pfu) per cell.

The plaque assay, combined with analysis of genetic homogeneity of viral stocks, was carried out as follows. Monolayers of BHK-21 cells were grown on 6-cm cell culture plates and infected with 20–30 pfu of collected viruses. After 1 h incubation at 37°C, the inoculum was removed, and cells were covered with GMEM containing 1% agarose and 1.5% FCS. At 48 h p.i. another layer of 1% agarose, containing neutral red staining solution, was added. 12 h later plaques were isolated. Virus was eluted with 0.5 ml GMEM and used to infect BHK-21 cells. Gluc activity in growth media was measured at 24 h p.i. as described above.

### Northern blotting and RT-PCR

Total RNA from collected virus stocks or cell lysates was extracted with TRIzol® reagent (Life Technologies) according to the manufacturer's protocol. For northern blotting, 5 µg of RNA was denatured in formamide-formaldehyde buffer at 65°C for 10 min followed by cooling on ice. Probes were separated by electrophoresis in a 1.4% agarose gel containing 0.2 M formaldehyde. RNA was transferred to Hybond- N+ filter (GE Healthcare) and fixed using UV Stratalinker 1800 (Stratagene). Digoxigenin (DIG)-labelled RNA detection probes complementary to the 3′ UTR of SFV4 and to β-actin mRNA were generated with a DIG hybridisation kit (Roche). Probes were hybridised overnight; blots were washed and developed according to the manufacturer's protocols.

For RT-PCR, cDNA was synthesised from extracted total RNA using a First Strand cDNA Synthesis Kit and random hexamer primers (Thermo Scientific). Sequences of interest were PCR-amplified from cDNA. DNA fragments were purified with a PCR purification kit (Genomed GmbH) and analysed by Sanger sequencing. Primer sequences are available upon request.

## Results

### miRNA target cassette design

Two different groups of miRNA targets were selected for insertion into DNA/RNA layered SFV replication-competent vectors. First, to analyse the effects of miRNAs on the rescue, multiplication and stability of recombinant viruses, target sites of ubiquitously expressed *Let-7*, *miR-17* and *miR-19*, previously used in VEEV vectors [28], were added to the cassettes (Fig. 1), hereafter referred as miR-cl1targets. Second, to construct vectors that could replicate in cervical carcinoma cells but not in normal cells, miR-cl2 target cassettes were designed. miR-cl2 cassettes contained the target sequences of *miR-214*, *miR-143d* and *miR-218*, which are down-regulated in human cervical cancer cells [43], [44], [45]. Each cassette contained two copies of three different miRNA target sequences, which were separated from each other by unspecific linkers of 8 nucleotides [46]. Cassettes were inserted in orientation allowing targeting of positive-strand viral RNAs, as targeting of negative-strand RNA has no detectable effect on alphavirus replication [28].

In contrast to previous studies using only target sequences fully complementary to selected miRNAs [28], [29], two additional approaches were tested in this study. First, nucleotides 9–12 of each miRNA target sequence were modified to prevent their pairing with the corresponding region of miRNA (miR-cl1S and miR-cl2S, referred as “sponge design”, Table 1). Second, the cassettes were constructed using naturally occurring miRNA target sequences (miR-cl1N and miR-cl2N, referred as “native design”, Table 1). The binding of miRNAs to sponge and native targets should result in translational silencing but not cleavage of targeted RNAs. As in previous studies, a classical miRNA target site design (miR-cl1P and miR-cl2P, referred to as “perfect design”) was also used. In this case, miRNA target sequences were fully complementary to the miRNA (Table 1); accordingly, the binding of miRNA to this target should result in cleavage of targeted RNAs.

### Cellular miRNAs suppress Gluc expression in a transient non-viral expression system

**Figure 2:**
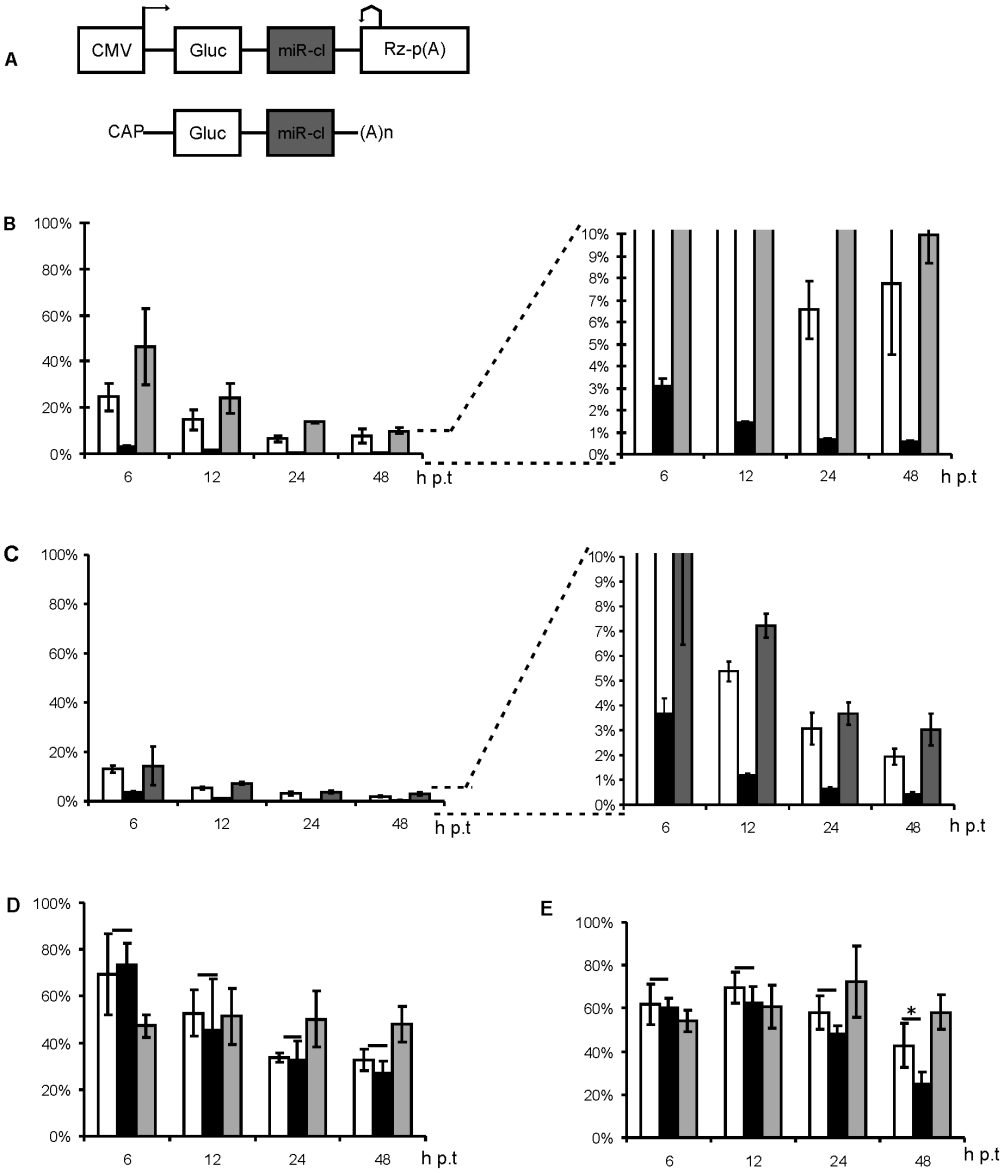
Insertion of miR-1cl and miR-cl2 cassettes suppresses Gluc expression from a non-viral vector. (A) Schematic representation of non-viral expression vector (above) and corresponding mRNA (below). The plasmid backbone of the vector is not shown. CMV, immediate-early promoter of human cytomegalovirus; Rz, negative strand ribozyme of hepatitis delta virus (cleavage site is indicated with bent arrow); p(A), early polyadenylation signal of simian virus 40; (A)n, poly(A) tail. The 5′ and 3′ UTRs of the transcribed mRNA correspond to those of SFV4. Effects of miR-cl1 target sequences on Gluc expression in transfected BHK-21 (B) or HeLa cells (C) and effects of miR-cl2 target sequences on Gluc expression in transfected BHK-21 (D) or HeLa cells (E) were analysed as follows. Cells were transfected by electroporation, and Gluc activity in the growth medium was measured at times indicated on the horizontal axes. The results were normalised to Gluc activity in the growth media of cells transfected with the Gluc expression vector lacking an miRNA target site; the corresponding activity was set at 100% (shown on vertical axes). In all panels, open, black and grey columns represent normalised data for sponge design, perfect design and native design cassettes, respectively. Each column represents an average of three independent experiments; error bars represent standard deviation. * designates a statistically significant difference (p<0.05) as determined by a Mann – Whitney U test.

The constructed targets were analysed for their ability to suppress gene expression in a non-viral expression system. For this analysis we used plasmids encoding Gluc; corresponding coding sequence was placed between 5′ and 3′ UTRs of SFV4 (Fig. 2A). In addition, all elements required for mRNA transcription and the body of expression plasmid were identical to those in pCMV-SFV4. Thus, the sequence context of target sites in non-viral and subsequently analyzed DNA/RNA layered SFV replication-competent vectors was identical. When the vectors containing miR-cl1 cassettes were transfected into BHK-21 cells, each of the three target designs caused clear and efficient repression of Gluc accumulation, as detected at 6 h p.t.; the effect increased with time (Fig. 2B). As expected, the extent of repression depended on the miRNA binding strategy. miR-cl1S and miR-cl1N targets resulted in approximately 10-fold reduction of Gluc activity (compared to non-targeted control vector) at 24 and 48 h p.t. (Fig. 2B). Expression levels for construct harbouring miR-cl1P target were approximately 200-fold reduced (24 and 48 h p.t.). In transfected HeLa cells the miR-cl1P target, again, caused the most prominent repression of Gluc accumulation; the magnitude of observed effect was very similar to that in BHK-21 cells (Fig. 2C). This data suggests that cleavage and degradation of targeted mRNA greatly contributes to the repression of marker expression. The effects caused by miR-cl1S and miR-cl1N targets in HeLa cells were indeed less pronounced. However, the repression caused by the presence of these sequences was two-to four-folds bigger than in BHK-21 cells (compare Fig. 2B and 2C).

miR-cl2 target sequences also reduced Gluc expression in transfected BHK-21 cells. While the effect increased over time, expression was reduced by only 2–3 fold at 48 h p.t. (Fig. 2D). Thus, miRNAs targeting the miR-cl2 cassette caused much less prominent inhibition than these targeting the miR-cl1 cassette. In clear contrast to miR-cl1P the miR-cl2P target failed to repress of Gluc expression more efficiently than the sponge design cassette and was only slightly more efficient than the native design cassette (Fig. 2D). When the miR-cl2 constructs were assayed in HeLa cells, the silencing of marker expression by different cassettes remained low up to 24 h p.t (Fig. 2E). These data are in line with reports that *miR-214*, *miR-143d* and *miR-218* are down-regulated in cervical cancer cells [43]–[45]; however the moderate effect may also result from the lower efficiencies of corresponding miRNAs. It was also observed that the marker expression was reduced nearly 5-fold by the miR-cl2P target by 48 h p.t (Fig. 2D); this repression was significantly (p = 0.0495) more efficient than that caused by insertion of the miR-cl2S cassette (Fig. 2E). In contrast to the cases with miR-cl1N and miR-cl1S, which were more efficient in HeLa cells, repression caused by miR-cl2N and miR-cl2S cassettes was similar in both cell types. Taken together, miR-cl2 cassettes were relatively poorly targeted both in HeLa and BHK-21 cells. These findings indicated that corresponding recombinant viruses could be rescued in BHK-21 and possibly also in HeLa cells.

### The miR-cl1 cassettes suppress rescue of recombinant virus from a DNA/RNA layered SFV vector

The generation of replication-competent virus from DNA/RNA layered SFV vectors is a multi-step process. First, the genome RNA of SFV is produced by cellular transcription machinery, transported to the cytoplasm and used for ns-protein expression. Next, viral genome replication involves synthesis of negative-strand RNA, formation of membrane-attached replicase complexes [47], [48] and is characterized by extremely efficient production of progeny RNA genomes and SG mRNAs. Finally, SFV infection leads to the complete shutdown of cellular transcription and translation, preventing further synthesis of miRNAs and components of the RNA-induced silencing complex (RISC). Importantly, the replication and subsequent spread of recombinant virus in cell culture is also accompanied by virus adaptation and selection [42].

First, the DNA/RNA layered replication-competent vector pCMV-SFV4-2SG-Gluc, which expresses Gluc from a duplicated SG promoter, was analysed. Using parental pCMV-SFV4 for comparison, we found that insertion of additional SG promoter and Gluc coding sequence did not affect the efficiency of infectious virus rescue from the cloned cDNA copy (hereafter term “infectivity” is used to designate this efficiency) or its growth characteristics (data not shown). Thus, these manipulations were well tolerated and pCMV-SFV4-2SG-Gluc represented a suitable tool to study the effects of miR-cl1 or miR-cl2 target insertions.

**Figure 3:**
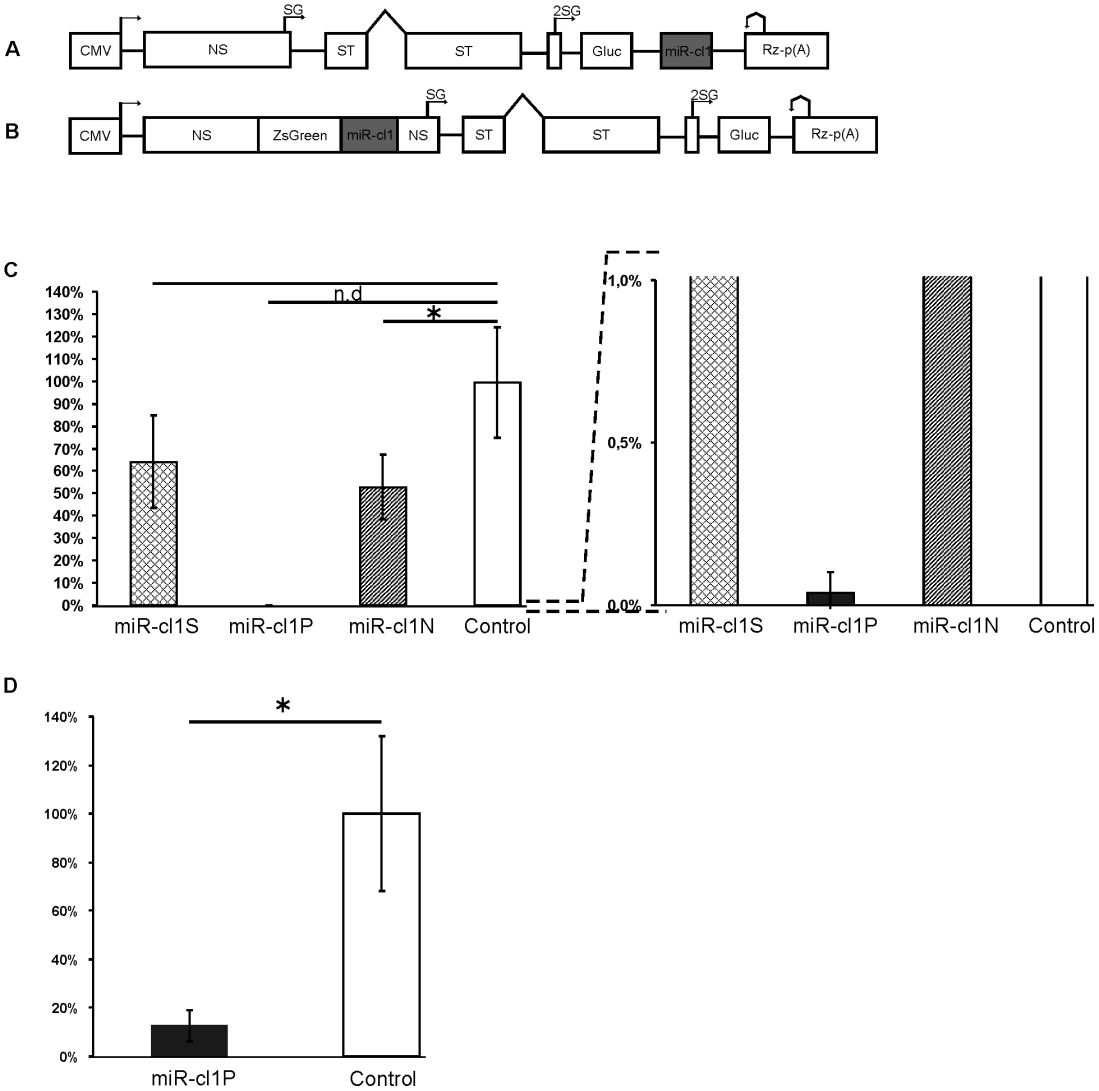
Infectivity of DNA/RNA layered SFV replication-competent vectors carrying miR-cl1 targets. (A). Schematic representation of the DNA/RNA layered replication-competent vector pCMV-SFV4-2SG-Gluc with an miR-cl1 target cassette in the 3′ UTR region. (B). Schematic representation of the DNA/RNA layered replication-competent vector pCMV-SFV(nsZsGreen-miR-cl1P)4-2SG-Gluc. (A, B). CMV, immediate-early promoter of human cytomegalovirus; Rz, negative strand ribozyme of hepatitis delta virus (cleavage site is indicated with bent arrow); p(A), early polyadenylation signal of simian virus 40; NS, ORF of ns-proteins; SG, subgenomic promoter of SFV (start site indicated with an arrow); 2SG, duplicated subgenomic promoter of SFV; ST, ORF of structural proteins. Symbol ∧ in the structural ORF designates an intron from the rabbit β-globin gene. miR-cl1 cassettes are shown in grey; sequences encoding Gluc and ZsGreen are indicated. The plasmid backbone of the vector is not shown; the indicated elements are not drawn to scale. (C). Infectivity of recombinant vectors with miR-cl1 target sites in their 3′ UTR regions, as measured by ICA. The vertical axes represent infectivity normalised to that of pCMV-SFV4-2SG-Gluc (designated as Control), which was taken as 100%. The miR-cl1 cassettes are indicated under the drawing. (D). Infectivity of recombinant vectors with or without miR-cl1 target in the ns-region, as measured by ICA. The vertical axes represent infectivity normalised to that of pCMV-SFV(nsZsGreen)4-2SG-Gluc (designated as Control), which was taken as 100%. The black column represents the infectivity of pCMV-SFV(nsZsGreen-miR-cl1P)4-2SG-Gluc. (C, D). Columns represent an average of three independent experiments; error bars represent standard deviation. * designate a statistically significant differences (p<0.05 as determined by a Mann – Whitney U test; n.d., not determined.

First the miR-cl1 targets were inserted immediately downstream of the Gluc coding sequence (Fig. 3A) and were therefore present in both recombinant virus genomes and viral SG mRNAs. ICA was used to reveal the effect of various miR-cl1 targets on recombinant virus rescue; the number of infection foci is proportional to infectivity of the DNA/RNA layered SFV replication-competent vector. It was found that in BHK-21 cells miR-cl1S and miR-cl1N targets resulted in less than 2-fold reduction of infectivity. In sharp contrast, infectivity of pCMV-SFV4-2SG-Gluc-miR-cl1P was drastically reduced (Fig. 3C); by rough estimation the infectivity of pCMV-SFV4-2SG-Gluc-miR-cl1P in BHK-21 cells was ∼2500-fold lower than that of pCMV-SFV4-2SG-Gluc. The infectivity was also very close to the detection limit of ICA. Not surprisingly, no plaques were obtained in some replicates of the experiment; therefore corresponding p-value could not be calculated (“zero” in ICA does not necessarily indicate the lack of infectivity; instead it indicates that the number of infection foci was <10). These data clearly indicate that, while the miR-cl1S and miR-cl1N target were quite efficient for suppressing marker protein expression in BHK-21 cells (Fig. 2B), their effect on recombinant virus rescue was minor. The most likely explanation for this result is that miRNA (RISC) bound to its target does not preclude viral RNA replication in BHK-21 cells.

Next, infectivity of pCMV-SFV4-2SG-Gluc-miR-cl1P, pCMV-SFV4-2SG-Gluc-miR-cl1S and pCMV-SFV4-2SG-Gluc-miR-cl1N was analyzed using HeLa cells. These cells are considerably less susceptible for SFV infection than BHK-21 cells. Coherently, the infectivity of pCMV-SFV4-2SG-Gluc was also found to be approximately 20-fold lower (∼5000 pfu/µg DNA in HeLa and ∼100,000 pfu/µg DNA in BHK-21 cells, respectively). No virus rescue was observed in pCMV-SFV4-2SG-Gluc-miR-cl1P transfected HeLa cells using ICA or any other assay. Thus, the insertion of miR-cl1P target reduced infectivity of this construct in HeLa cells at least 5000-fold. Furthermore, it was observed that the insertion of miR-cl1S and miR-cl1N targets diminished the infectivity of corresponding DNA/RNA layered SFV replication-competent vectors in HeLa cells ∼200-fold. Again, more accurate determination of the extent of the repression was not possible as the infectivity's of these constructs were very close to the detection limit of ICA. Thus, in contrast to BHK-21 cells, all miR-cl1 targets were highly efficient in HeLa cells. For pCMV-SFV4-2SG-Gluc-miR-cl1S and pCMV-SFV4-2SG-Gluc-miR-cl1N this may represent a consequence of more efficient repression of targeted mRNA translation (Fig. 2C) and/or it may originate from less robust replication of SFV in HeLa cells which makes it more sensitive to the effects of different inhibitors.

Insertion of *miR-124* target sites into the SFV4 ns-region inhibits virus replication in neuronal cells where the corresponding miRNAs are expressed [29]. In this case, the virus has miRNA targets only in genomic but not SG mRNAs; the non-replicating SG mRNA cannot act as decoy targets to reduce the efficiency of miRNA-mediated repression. To compare the efficacy of this approach with the one described above, the miR-cl1P cassette was inserted into the ns-region of a DNA/RNA layered SFV replication-competent vector. To avoid insertion of amino acid residues, encoded by the target, to the viral ns-proteins, miR-cl1 target sequences were added to the ZsGreen coding sequence (Fig. 3B), which was then inserted into an SFV vector using the previously described approach [15]. Thus, recombinant viruses rescued from pCMV-SFV(nsZsGreen)4-2SG-Gluc and pCMV-SFV(nsZsGreen-miR-cl1P)4-2SG-Gluc express normal viral proteins and easily detectable ZsGreen and Gluc markers (Fig. 3B). As measured by ICA, the infectivity of pCMV-SFV(nsZsGreen)4-2SG-Gluc in BHK-21 cells was reduced approximately 200-fold from that of pCMV-SFV4 or pCMV-SFV4-2SG-Gluc. This result may have been due to the substantial increase in the recombinant genome size (inserted sequences accounted for >1600 b or >15% of original genome size of SFV4), resulting in slower replication and reduced packaging efficiency [42]. Due the low infectivity the effect of miR-cl1P target site insertion was analysed only in BHK-21 cells. It was observed that the insertion of the miR-cl1P target site resulted in an additional 7-fold drop in infectivity (Fig. 3D). Thus, the effect of miR-cl1P site insertion into the ns-region was significant (p = 0,495) but still more than 300-fold less prominent than that observed for its insertion into the SFV 3′ UTR (compare Fig. 3C and 3D), confirming the latter as the clearly preferred region for miRNA target insertion. Both viruses carrying coding sequences of ZsGreen and Gluc markers were also genetically unstable; foreign sequences in the ns-region were rapidly lost regardless of the presence of an miR-cl1P target site. Due to the relatively minor effect of miR-cl1P insertion on the rescue efficiency and the extreme genetic instability of rescued viruses, pCMV-SFV(nsZsGreen)4-2SG-Gluc and pCMV-SFV(nsZsGreen-miR-cl1P)4-2SG-Gluc were excluded from further analysis.

### The miR-cl1P target causes contra-selection and results in genetic instability in recombinant viruses

The presence of a miR-cl1P target inhibited recombinant virus rescue in BHK-21 cells (Fig. 3C) and completely blocked it in HeLa cells (no virion production or virus-mediated Gluc expression could be detected). By this reason following experiments with recombinant vectors, carrying miR-cl1P target site, were preformed only in BHK-21 cells. First, it was important to compare the growth characteristics of viruses rescued from pCMV-SFV4-2SG-Gluc and pCMV-SFV4-2SG-Gluc-miR-cl1P. As it was anticipated that cellular miRNAs would suppress the growth of the targeted virus, the following experiments were carried out either in the presence of 2-O-Met RNA oligonucleotides antisense to *Let-7*, *miR-17* and *miR-19* (miRNA inhibitors) or in the presence of an irrelevant 2-O-Met RNA control oligonucleotide.

**Figure 4:**
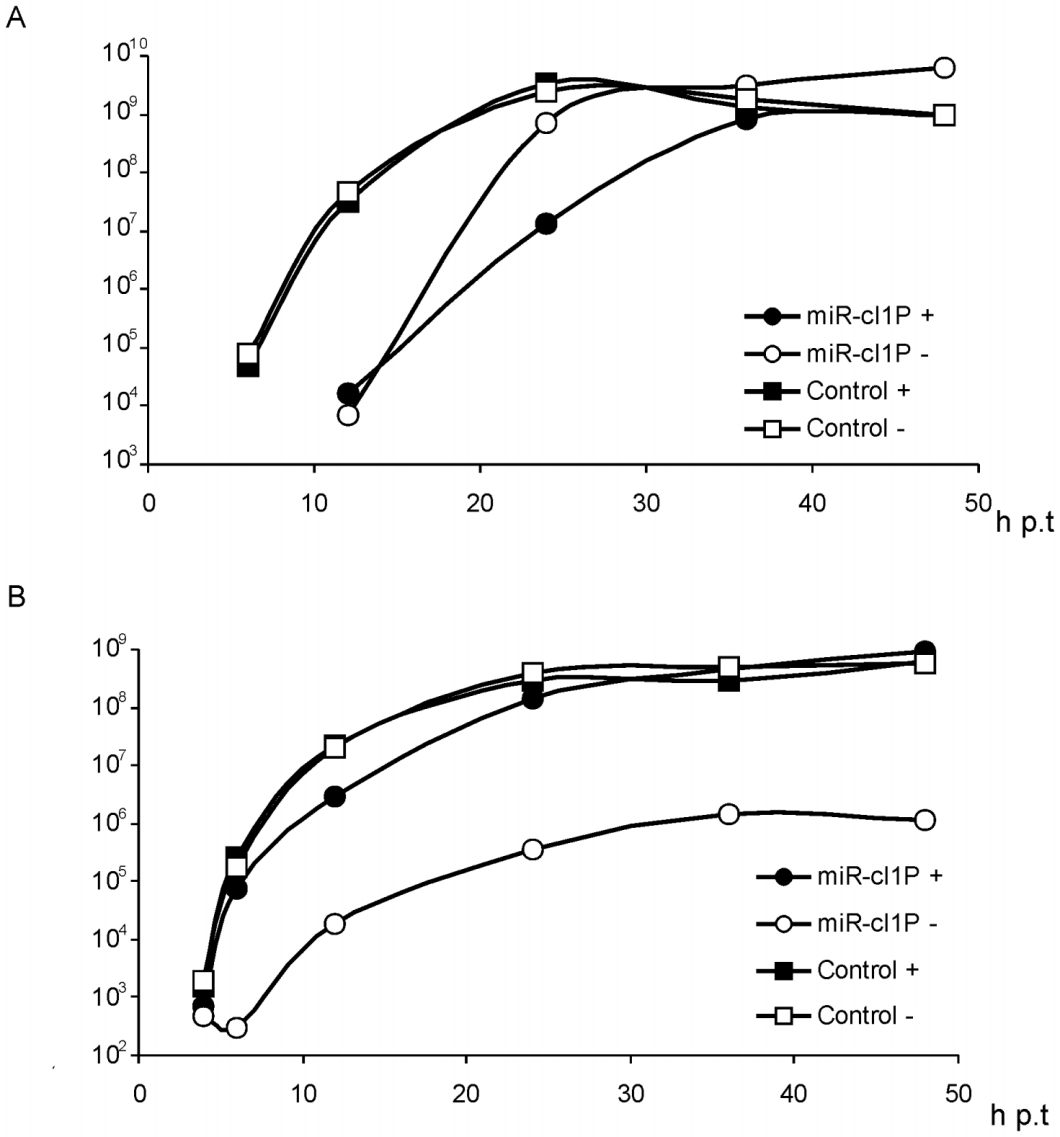
Effects of miRNA inhibitors on the rescue, multiplication and Gluc expression of recombinant vectors. BHK-21 cells were electroporated with 1 µg pCMV-SFV4-2SG-Gluc (Control) or pCMV-SFV4-2SG-Gluc-miR-cl1P (miR-cl1P) in the presence of 2-0-Met-RNA oligonucleotides complementary to *Let-7*, *miR-17* and *miR-19* (300 pmol of each; indicated with black symbols and “+”) or in the presence of 900 pmol irrelevant control oligonucleotide (indicated with open symbols and “−”). Titres of rescued viruses and Gluc activity in growth media were monitored up to 48 h p.t. (horizontal axes). (A) Titres of rescued recombinant viruses. Vertical axes represent the virus titre (pfu/ml) in the growth medium. (B) Expression of Gluc by recombinant viruses. Vertical axes represent the Gluc activity (in relative luciferase activity units) in the growth medium. Representative data from one from three reproducible experiments is shown in each panel.

When BHK-21 cells were electroporated with pCMV-SFV4-2SG-Gluc, recombinant virus was detectable as early as 6 h p.t.; the virus stock titre increased rapidly and reached a maximal value by 24 h p.t. (Fig. 4A). Similarly, high levels of Gluc activity were detected at 6 h p.t. and reached its maximal level by 24 h p.t. (Fig. 4B). Virus growth and Gluc activity were not affected by miRNA inhibitors, indicating that their presence did not affect rescue and multiplication of replication-competent SFV. The rescue of recombinant virus from pCMV-SFV4-2SG-Gluc-miR-cl1P was detectable only after 12 h p.t.. In the presence of miRNA inhibitors, the virus grew slower and reached lower titres than in the presence of an irrelevant control oligonucleotide (Fig. 4A). Notably, the opposite was true for marker expression: high levels of Gluc activity, similar with those of control viruses, were detected only in the presence of miRNA inhibitors (Fig. 4B).

**Figure 5:**
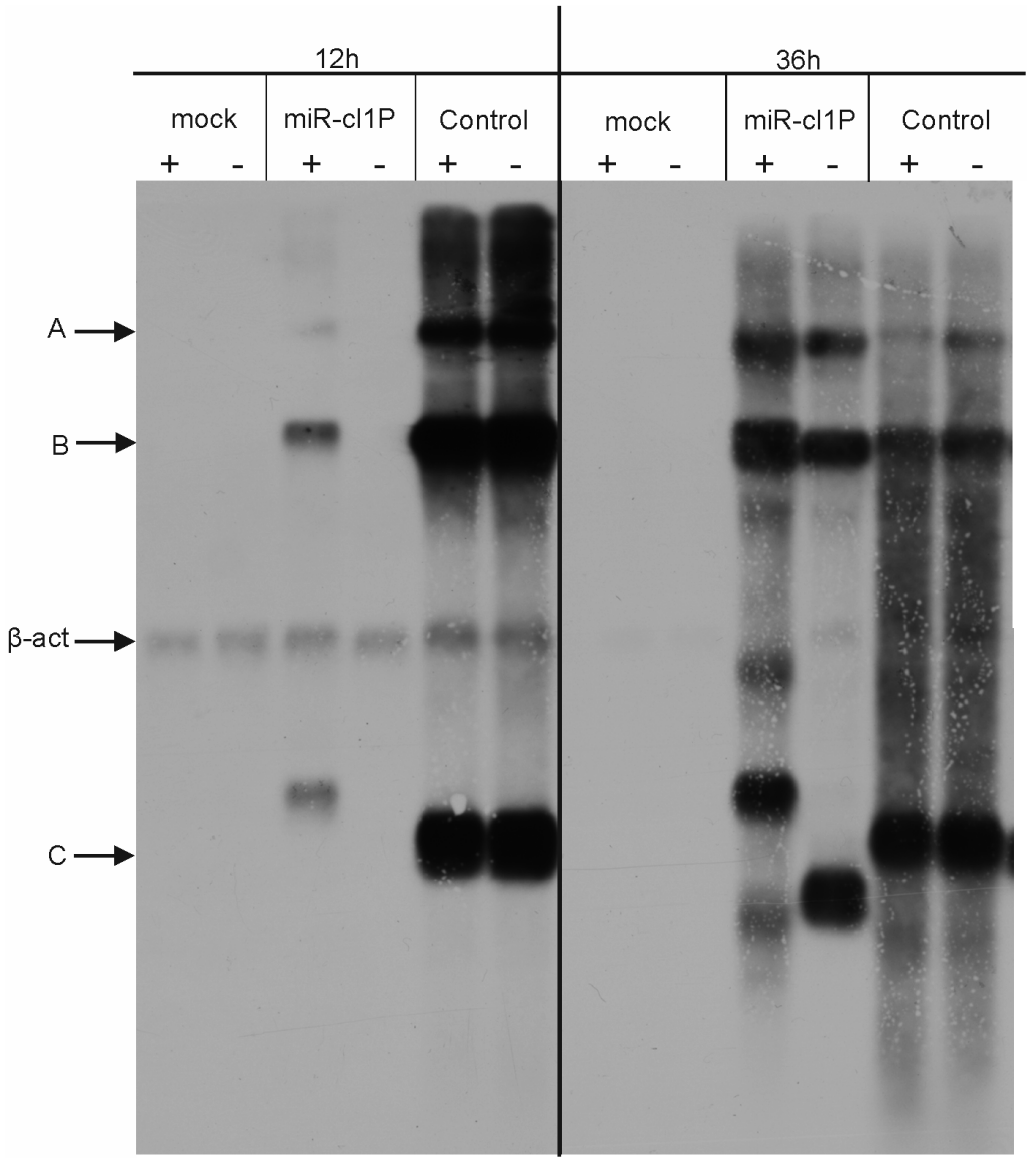
Northern blot analysis of SFV RNAs in transfected cells. BHK-21 cells were transfected with 1 µg pCMV-SFV4-2SG-Gluc (Control) or pCMV-SFV4-2SG-Gluc-miR-cl1P (miR-cl1) in the presence of 300 picomol of each miRNA inhibitors (designated with “+”) or 900 pmol of irrelevant control oligonucleotide (designated with “−”). Total RNA was isolated from cells at 12 h p.t. or 36 h p.t.. RNA (5 µg) was loaded on a 1.4% denaturing agarose gel, resolved by electrophoresis and visualised by northern blotting with a DIG-labelled RNA probe complementary to the 3′ UTR of SFV4 or to β-actin mRNA. Arrows left of the panel designate viral genomic RNA (A), SG mRNA made from the native SG promoter (B), additional SG RNA synthesised from the duplicated SG promoter in SFV4-2SG-Gluc (C) and β-actin mRNA, used as loading control (β-act). The panel is composed of pictures obtained by two different exposures of the same filter, which was necessary due to the huge difference in viral RNAs levels at 12 and 36 h p.t.. After the shorter exposition (36 h p.t.), mRNA of β-actin is hardly detectable. Longer exposure (12 h p.t.) detects mRNA of β-actin in mock-samples at 36 h. p.t., but it is masked by the strong signal from viral RNAs in samples from cells transfected with DNA/RNA layered SFV replication-competent vectors. The experiment was repeated twice with similar results.

The straightforward explanation for the dynamics of virion and Gluc accumulation is the following: intact virus genomes rescued from pCMV-SFV4-2SG-Gluc-miR-cl1P were able to replicate only in the presence of miRNA inhibitors. In their absence, the region encoding Gluc and presumably also the miR-cl1P target was deleted, and the original viruses were rapidly outcompeted by aberrant ones. To verify this hypothesis, total RNA from transfected cells was collected at 12 h and 36 h p.t. and analysed by northern blotting. This assay confirmed that virus rescued from pCMV-SFV4-2SG-Gluc was genetically stable (Fig. 5). Consistent with their delayed rescue (Fig. 4A), viral RNAs carrying an miR-cl1P target accumulated at a reduced rate. Importantly, in the absence of miRNA inhibitors, the RNAs of targeted virus were undetectable at 12 h p.t.; in contrast, all expected viral RNAs were detected in samples obtained from cells co-transfected with mRNA inhibitors (Fig. 5). At 36 h p.t., however, RNAs of virus rescued from pCMV-SFV4-2SG-Gluc-miR-cl1P were abundant both in cells co-transfected with miRNA inhibitors or a control oligonucleotide. However, three RNAs with expected sizes were detected only in the samples obtained from cells co-transfected with miRNA inhibitors. In addition to these, at least three additional viral SG mRNAs were detected (Fig. 5). This finding clearly indicated on-going genetic re-arrangements, most likely involving deletions in the miR-cl1P target region. For the cells co-transfected with irrelevant control oligonucleotides, the expected duplicated SG mRNA was not detected; instead the major additional SG mRNA was smaller than the predicted size (Fig. 5). As this virus was also unable to highly express Gluc (Fig. 4B), the obvious conclusion is that the observed deletion involved at least some sequences encoding this enzyme.

To reveal the exact nature of these aberrations, the genomes of recombinant viruses were isolated from culture media at 12 h and 36 h p.t. and analysed by RT-PCR and sequencing. This analysis confirmed the formation of recombinant genomes with deletions in the miR-cl1P target site region. Furthermore, the same tendency as it was revealed by northern blot was observed: the loss of miR-cl1P target sequence occurred more quickly and was virtually complete in the absence of miRNA inhibitors. Though the exact positions of deletions varied, the miR-cl1P target was always completely removed in addition to a variable portion of sequence encoding the C-terminal region of Gluc. Taken together, northern blot and RT-PCR/sequencing analysis confirmed that virus rescued from pCMV-SFV4-2SG-Gluc-miR-cl1P was strongly attenuated and therefore genetically unstable. The miRNA inhibitors enabled replication of these genomes and slowed down, but did not prevent, genetic rearrangements leading to the loss of the miR-cl1P target region.

### miR-cl2P cassette has a mild effect on the rescue of recombinant SFV from a DNA/RNA layered vector

**Figure 6:**
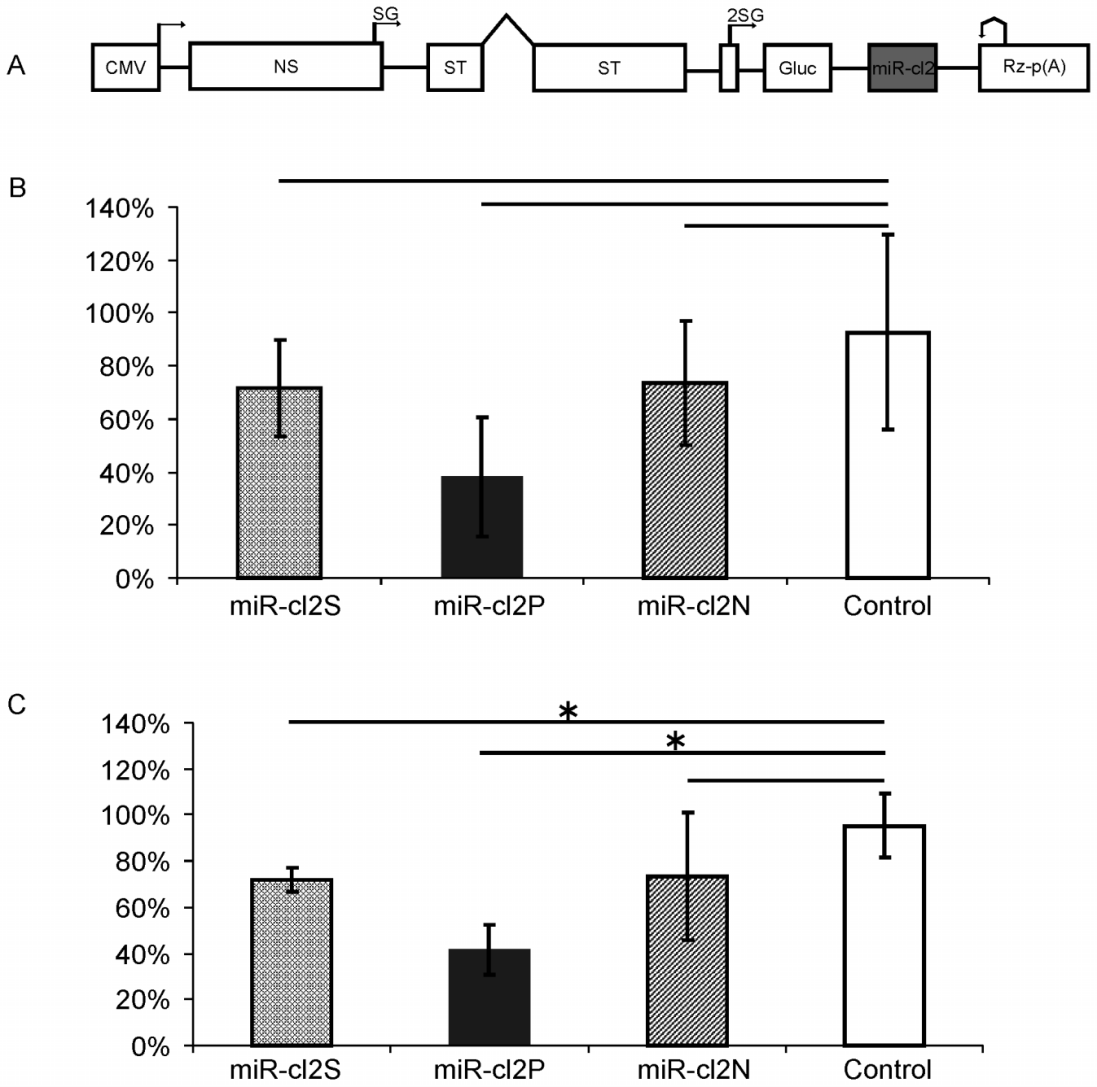
Infectivity of DNA/RNA layered SFV replication-competent vectors carrying miR-cl2 targets. (A) General design of the vectors; the same symbols as in Fig. 3A are used to designate different sequences. (B, C) Infectivity of recombinant vectors carrying different miR-cl2 targets was measured by ICA in BHK-21 (B) and HeLa (C) cells. The vertical axes represent infectivity normalised to that of pCMV-SFV4-2SG-Gluc (designated as Control), which was taken as 100%. The inserted miR-cl2 cassettes are indicated under the drawing. Columns on the graph represent an average of three independent experiments; error bars represent standard deviation. * designates a statistically significant difference (p<0.05), as determined by a Mann – Whitney U test.

The results obtained using non-viral expression vectors (Fig. 2D, 2E) suggested that the effects of miR-cl2 targets on the infectivity of DNA/RNA layered SFV replication-competent vectors could be small. Consistently, the results of ICA in BHK-21 cells revealed that though all miR-cl2 targets reduced the infectivity of DNA/RNA layered SFV vectors the effect did not reach statistical significance even for pCMV-SFV4-2SG-Gluc-miR-cl2P (p = 0.081) (Fig. 6B). miR-cl2N target failed to significantly reduce infectious virus rescue also in HeLa cells (Fig. 6C). In contrast, in HeLa cells the infectivity of both pCMV-SFV4-2SG-Gluc-miR-cl2S and pCMV-SFV4-2SG-Gluc-miR-cl2P was significantly lower (p = 0.0495) than that of pCMV-SFV4-2SG-Gluc (Fig. 6C). As in case of pCMV-SFV4-2SG-Gluc-miR-cl1S and pCMV-SFV4-2SG-Gluc-miR-cl1N vectors, the more prominent repression in HeLa cells may originate from less robust SFV replication. The 3.5-fold reduction of infectivity, observed for pCMV-SFV4-2SG-Gluc-miR-cl2P in HeLa cells was, however, much smaller than that caused by the presence of a any of miR-cl1 target sites. This finding is consistent with the low abundance/activity of *miR-214*, *miR-143d* and *miR-218* in HeLa cells and/or may result from the lower efficiency of these miRNAs (compared to miRNAs targeting miR-cl1 cassette).

### The miR-cl2P target increases recombinant SFV genome sensitivity to *miR-214* expression but also results in genetic instability

The results of previous experiments prompted the hypothesis that virus rescued from pCMV-SFV4-2SG-Gluc-miR-cl2P should infect and multiply in HeLa cells but may be restricted in cells expressing larger amounts of *miR-214*, *miR-143d* and/or *miR-218*. To verify this directly attempts to construct HeLa cell lines stably expressing increased amounts of these miRNAs were made. These attempts were, however, only partly successful and only a cell line over-expressing *miR-214* (HeLa-miR214) was obtained for further analysis.

**Figure 7:**
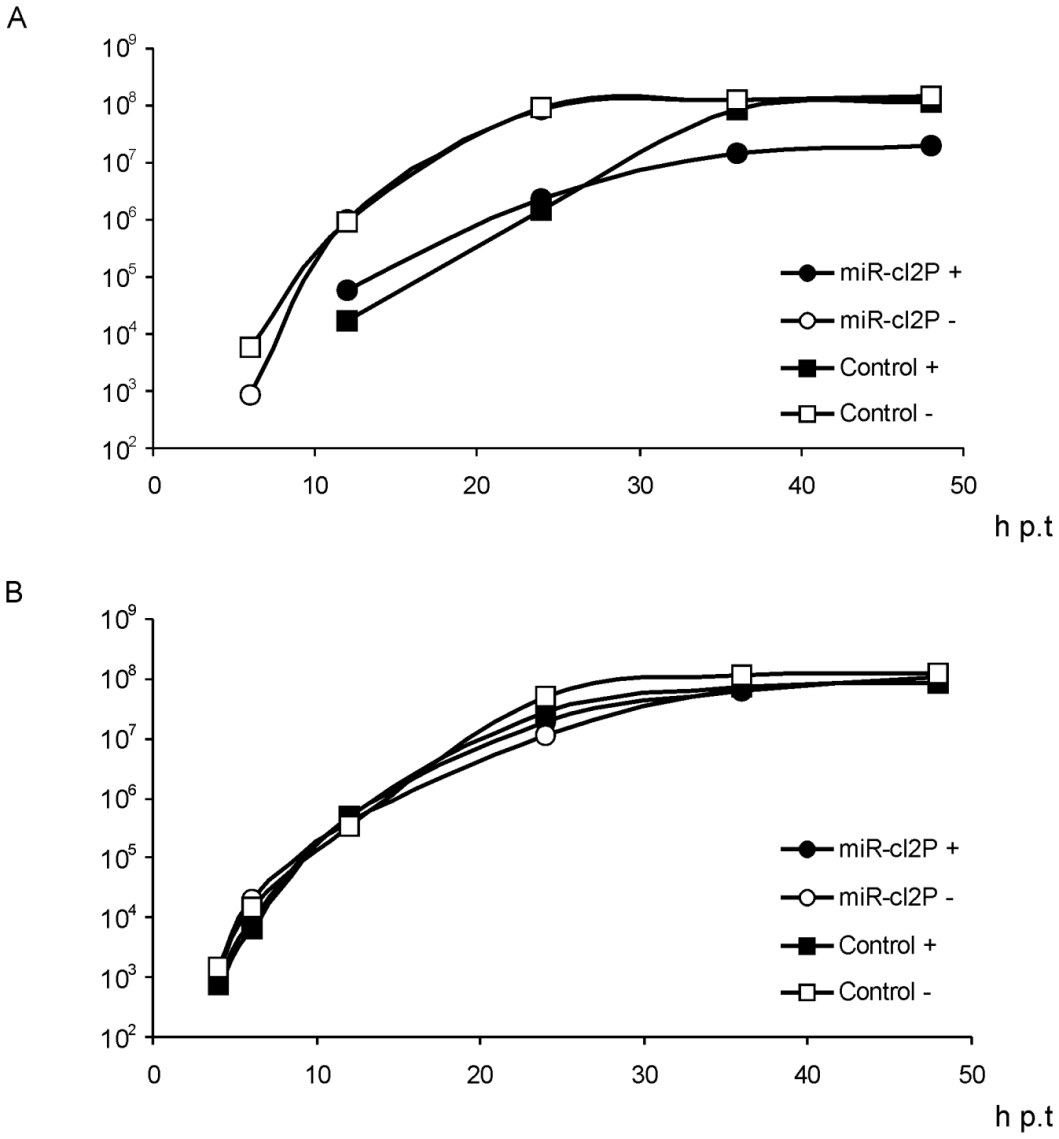
Effect of *miR-214* over-expression on the rescue, multiplication and Gluc expression of recombinant vectors. Normal HeLa cells (open symbols and “−”) and HeLa-miR214 cells (black symbols and “+”) were electroporated with 1 µg pCMV-SFV4-2SG-Gluc (designated as Control) or pCMV-SFV4-2SG-Gluc-miR-cl2P (designated as miR-cl2P). Titres of rescued viruses and Gluc activity in growth media were monitored up to 48 h p.t. (horizontal axes). (A) Growth of rescued recombinant viruses. Vertical axes represent the virus titre (pfu/ml) in the growth medium. (B) Expression of Gluc by recombinant viruses. Vertical axes represent the Gluc activity (in relative luciferase activity units) in the growth medium. Representative data from one of three reproducible experiments is shown in each panel.

Analysis of the rescue, growth and marker expression by pCMV-SFV4-2SG-Gluc in HeLa and HeLa-miR214 cells revealed an unexpected effect. The rescue and maximal titres of the control virus in HeLa-miR214 cells were both delayed by 6–12 h (Fig. 7A). Interestingly, this delay did not affect Gluc expression, which was identical to that in HeLa cells (Fig. 7B). The reasons behind this reproducible effect were beyond the scope of this study. It can be speculated, however, that over-expression of *miR-214* changed the expression of cellular factors that were required for efficient virion formation but dispensable for SFV gene expression. Indeed, as it has been shown for mutants of VEEV, viral protein expression and the efficiency of virion formation are not necessarily strictly coupled [49]. Similarly, over-expression of *miR-214* had little or no effect on Gluc expression by virus rescued from pCMV-SFV4-2SG-Gluc-miR-cl2P (Fig. 7B). In contrast, *miR-214* clearly affected growth of the targeted virus. Accumulation of its virions in HeLa-miR214 cell culture was further delayed, and in contrast to the control virus, its maximal titres failed to reach the same levels as in normal HeLa cells. Thus, it was confirmed that the miR-cl2P target site has little effect on virus replication in HeLa cells but reduces virus production in cells expressing large amounts of *miR-214*.

The preferential growth of virus rescued from pCMV-SFV4-2SG-Gluc-miR-cl2P in cells with low levels of *miR-214* expression indicates that this approach can be potentially used for construction of recombinant alphaviruses that replicate specifically in cancer cells. To analyze the genetic stability of rescued virus it was passaged twice in HeLa cells at an MOI of 0.1 pfu/cell and the genetic homogeneity of the obtained stock was analysed first by plaque purification and Gluc activity assay. This analysis revealed that all 24 plaques selected for the analysis were formed by viruses expressing Gluc, indicating high genetic stability. To identify the presence of genomes lacking the miR-cl2P target site but not the Gluc reporter gene or the viruses with titres too low for detection in plaque assay analysis, genetic stability was also analysed by RT-PCR. In addition to the RT-PCR products corresponding to the constructed recombinant viruses, aberrant genomes with deletions in the miR-cl2P target region were found to be present in the initial stock obtained from HeLa cells transfected with pCMV-SFV4-2SG-Gluc-miR-cl2P as well as in virus stocks obtained by passaging virus on HeLa cells. Thus, the small growth disadvantage of viruses with a miR-cl2P target site, detected by both ICA (Fig. 6C) and growth curve analysis (Fig. 7A, 6 h p.t. time point), was sufficient to generate selective pressure against genomes targeted by *miR-214*, *miP-218* and *miR143d*. The effect was much less prominent than in viruses rescued from pCMV-SFV4-2SG-Gluc-miR-cl1P; consistently, the targeted genomes were not rapidly outcompeted by aberrant ones. Nonetheless, these data suggest that genetic instability is an intrinsic property of miR-targeted alphavirus genomes.

## Discussion

Control of vector derived-gene expression is often the most challenging aspect in the design and application of different anti-cancer and gene therapy vectors. It is especially important for virus-based vectors that are potentially dangerous infectious agents. As DNA regulatory elements cannot be used to control viruses with RNA genomes, regulation of the corresponding vectors has been particularly challenging. The discovery of miRNAs and their role in post-transcriptional regulation of gene expression has provided researchers with new and efficient tools to achieve control over these vectors. Though RNA viruses can be designed to express siRNA or miRNA precursors, the most popular approach to regulate their replication and/or spread has been to engineer their genomes and/or mRNAs such that they become targets of pre-existing cellular miRNAs. This approach is advantageous because the miRNA pools in different cells types differ from each other. Furthermore, natural RNA viruses are known to use differences in cellular miRNA pools to generate cell type or tissue specificity. Thus, the liver-specific *miR-122* is an important co-factor for hepatitis C virus [50]. Conversely, organisms use miRNAs to restrict viruses such as enterovirus 71 [51]. Similarly, the artificially designed strategies use miRNA targeting to restrict RNA virus infection. This strategy, however, inevitably generates selection pressure that forces viruses to eliminate the inserted regulatory sequences. Thus, the optimal design of an miRNA target site, the level and mechanism of repression sufficient and necessary to control the infection of recombinant virus, the strategy of miRNA binding to its target and selection of the optimal genomic location of the target site are important. For most viral vectors, the impact and significance of these factors have not been systematically studied. We used DNA/RNA layered SFV replication-competent vectors to demonstrate the benefits and limitations of different miRNA targets and their insertion sites.

When designing target sites for cellular miRNAs in RNA virus genomes, the first questions are: what target sites should be used? How many copies of each target are needed? How should they be combined with each other? To our knowledge, there is no universal algorithm that provides easy answers to these questions. In this study, we used previously described design of miRNA target cassettes that consisted of two copies of three different miRNA targets (Fig. 1). The effects caused by the insertion of these cassettes were analysed using both non-replicating vectors and DNA/RNA layered SFV replication-competent vectors. In the absence of RNA replication, the targets containing binding sites for ubiquitously expressed *let-7*, *miR-17* and *miR-19* were most efficient if they matched perfectly with cellular miRNA sequences. Targets containing sponge and native binding sites also reduced Gluc already at very early time points (Fig. 2B, 2C). When the same targets were analysed in the context of replication-competent vectors, constructs infectivity in BHK-21 cells was drastically reduced only when the targets were located in the 3′ UTR of the recombinant viral genome and were fully complementary to cellular miRNAs (Fig. 3C). This finding indicates that in these cells, where SFV replication is very efficient and robust, cleavage of the targeted RNA genome was required to inhibit virus replication. In contrast, for HeLa cells where both SFV rescue and its subsequent multiplication are less efficient (such effect can also be seen from reduced final titres of control viruses in HeLa cells, compare Fig. 4A and Fig. 7A), the sponge- and native design miR-cl1 targets efficiently suppressed infectious virus rescue. Thus, the requirements for suppression of recombinant virus rescue are clearly different for different cell lines; furthermore, correlation between suppression of infectious virus rescue and silencing of translation from non-replicating mRNA was not absolute.

The most obvious difference in silencing of recombinant virus rescue and suppression of translation from non-replicating mRNA lies in their different nature. The silencing of translation is a quantitative effect, while the silencing of virus rescue has a more qualitative nature: a single infectious viral RNA genome can result in viral replication. Accordingly, after the infectious RNA has been transcribed from cDNA and transported to cytoplasm, there is little time for miRNA targeting to destroy or inactivate it; once the ns-proteins are synthesised and the viral replicase complex formed, the RNA genome is most likely poorly, if at all, susceptible to miRNA-mediated inhibition. Furthermore, the subsequent RNA replication produces new positive strands in quantities that may preclude their suppression by the existing pool of miRNAs. Therefore, RNA cleavage—leading to the destruction of the template and/or physical separation of elements required for initiation of replication—becomes essential. However, as it is evident from the profound effects of miR-cl1S and miR-cl1N target sites on the recombinant virus rescue in HeLa cells, this requirement is not absolute. The reasons why the relatively small (two to four-fold) difference in suppression of targeted mRNA translation in is converted into much bigger (∼100-fold) difference in infectivity of corresponding DNA/RNA layered SFV replication competent vectors are unclear and additional studies are required. It seems, however, unlikely that some threshold level of inhibition of mRNA translation must be reached in order to efficiently suppress the infectivity of DNA/RNA layered SFV vectors; results with vectors carrying miR-cl2S or miR-cl2P sites (Fig. 6C) also argue against this possibility. Given the large number of known mechanisms of miRNA mediated repression of translation it is possible that the suppression of virus rescue originates from combination different mechanisms. Interestingly, we have found that in HeLa cells, increased amounts of several cellular proteins, found also in stress granules and processing bodies or involved in RNA translation, are present in replication organelles of SFV [52]; the set of these proteins is not necessarily same in different cell types. It is therefore possible that the effects caused by binding of miRNAs (RISC) to viral RNA genome may also be, at least in part, be cell-type specific. It is also possible that the requirements of replication complex formation (again, host factor are known or suspected to take part in this process) are somewhat different in different cell types. This may also result in different susceptibility of viral genome to the inhibitory effects of miRNAs.

Because the distance between the 3′ UTR and ns-protein ORF is great, this region may not be the most efficient site for miRNA target insertion. However, it was shown that insertion of miRNA target sites in the VEEV 5′ UTR did not lead to better inhibition [28]. Nevertheless, *miR-124* targets in the ns-region of the SFV genome are efficient [29]. Our head-to-head comparison of these approaches unequivocally demonstrated that the miRNA target (or at least a target that promotes cleavage of viral RNA) located in the 3′ UTR is dramatically more efficient than the same target located in the ns-region (compare Fig. 3C and 3D). This result may be a consequence of the fact that in cellular mRNAs, the miRNA targets are usually located in 3′ UTR regions and/or that active translation of the ns-region interferes with miRNA binding to the same region.

The prominent inhibition of recombinant virus rescue serves as a good indication of targeted virus suppression in corresponding cells. However, as most RNA viruses lack proofreading activity and are characterised by high levels of RNA recombination, the genetic instability of recombinant viral genomes should not be overlooked. While it may not be important for replicon vectors, which are capable of only a single round of replication [28], stability is clearly important for replication-competent vectors and recombinant viruses. Unfavourable changes such as genome enlargement [42] or point mutations affecting RNA replication [53], [54] result in the rapid appearance of mutants lacking these disadvantages. Generally, the growth disadvantage of the original mutant virus is inversely proportional to the time required for viruses with adaptations to out-compete their progenitor; during strong contra-selection, this transition usually happens within a few hours (our repeated observation). These tendencies were clearly observed for viruses rescued from pCMV-SFV4-2SG-Gluc-miR-cl1P and pCMV-SFV4-2SG-Gluc-miR-cl2P. In the case of the former, this behaviour was expected, as the repression of rescue of these viruses by miRNAs was immense (Fig. 3C). The extremely efficient suppression of rescue and replication by miRNAs was also the most likely reason why even specific miRNA inhibitors could not completely prevent deletions in the target site region. In contrast, plaque assays revealed that the genomes of recombinant vectors containing miR-cl2P targets were rather stable. However, a more sensitive RT-PCR method revealed that even the relatively inefficient miR-cl2P target caused instability and the rapid appearance of genomes without the target site. The obvious conclusion from these observations is that the strategy used to limit the recombinant virus growth to the cancer cells cannot rely exclusively on miRNA targeting or, at very least, requires approaches that prevent target elimination. Therefore, miRNA targets should be engineered from naturally existing coding sequences by silent mutagenesis and/or should be inserted in several loci of virus genomes instead of combining all targets into a single cluster.

What could be perspectives of the use of miRNAs to control recombinant alphavirus rescue, infection and spread? The strategy used to increase the biosafety of VEEV replicon vectors [28] can obviously be used for other alphavirus-based replicon vectors as well. For these applications, “the stronger the inhibition, the better the outcome” applies. In contrast, our results indicate that the main hurdle for miRNA-mediated restriction of replication-competent alphavirus vectors is not suppression of virus replication but stabilisation of targeted viral genomes. Achieving of suitable balance between miRNA-mediated control and genetic stability of recombinant viruses requires further investigations. The designs already used to construct miRNA-regulated alphavirus vectors could, however, be used to prevent spreading of infection to organs and tissues associated with viral pathogenesis. Indeed, the incorporation of *miR-124* targets made SFV non-neurovirulent in mice [29]. Furthermore, *in vivo* experiments showed that most viral progeny (89%) maintained the original miRNA target sequence after intraperitoneal delivery and passage in animals. This observation indicates that the “keep out” strategy is less prone to cause the elimination of miRNA target sequences. However, the possibility that the loss of miRNA targets *in vivo* is intrinsically slower than in *in vitro* cell culture experiments, cannot be excluded. Indeed, this phenomenon has been observed in alphaviruses containing marker genes [15]. Thus, other viruses with miRNA target sites could also be more efficient and stable *in vivo*. Furthermore, miRNA targeting could be used to limit replication of arboviruses in their arthropod vectors. For this mosquito-specific miRNA target sites could be introduced as a cluster to the 3′UTR region of the alphavirus genome.
